# Supplementary material for: A review of advancements in the theory and characterization of soil macropore structure
Source: PeerJ. 2024 Nov 14;12:e18442. doi: 10.7717/peerj.18442 (PMC11569784; doi:10.7717/peerj.18442)
Supplement: Supplemental Information 1 [file peerj-12-18442-s001.docx]

**Table High-frequency country and frequencies for CT characterization of soil macropore studies.**

| Countries | Frequencies |
| --- | --- |
| Peoples R China | 107 |
| USA | 62 |
| England | 35 |
| Germany | 27 |
| Brazil | 19 |
| Denmark | 18 |
| Spain | 17 |
| Canada | 16 |
| Scotland | 12 |
| Russia | 12 |
| France | 11 |
| Australia | 11 |
| Sweden | 10 |
