# Supplementary figures and images for: A review of advancements in the theory and characterization of soil macropore structure
Source: PeerJ. 2024 Nov 14;12:e18442. doi: 10.7717/peerj.18442 (PMC11569784; doi:10.7717/peerj.18442)

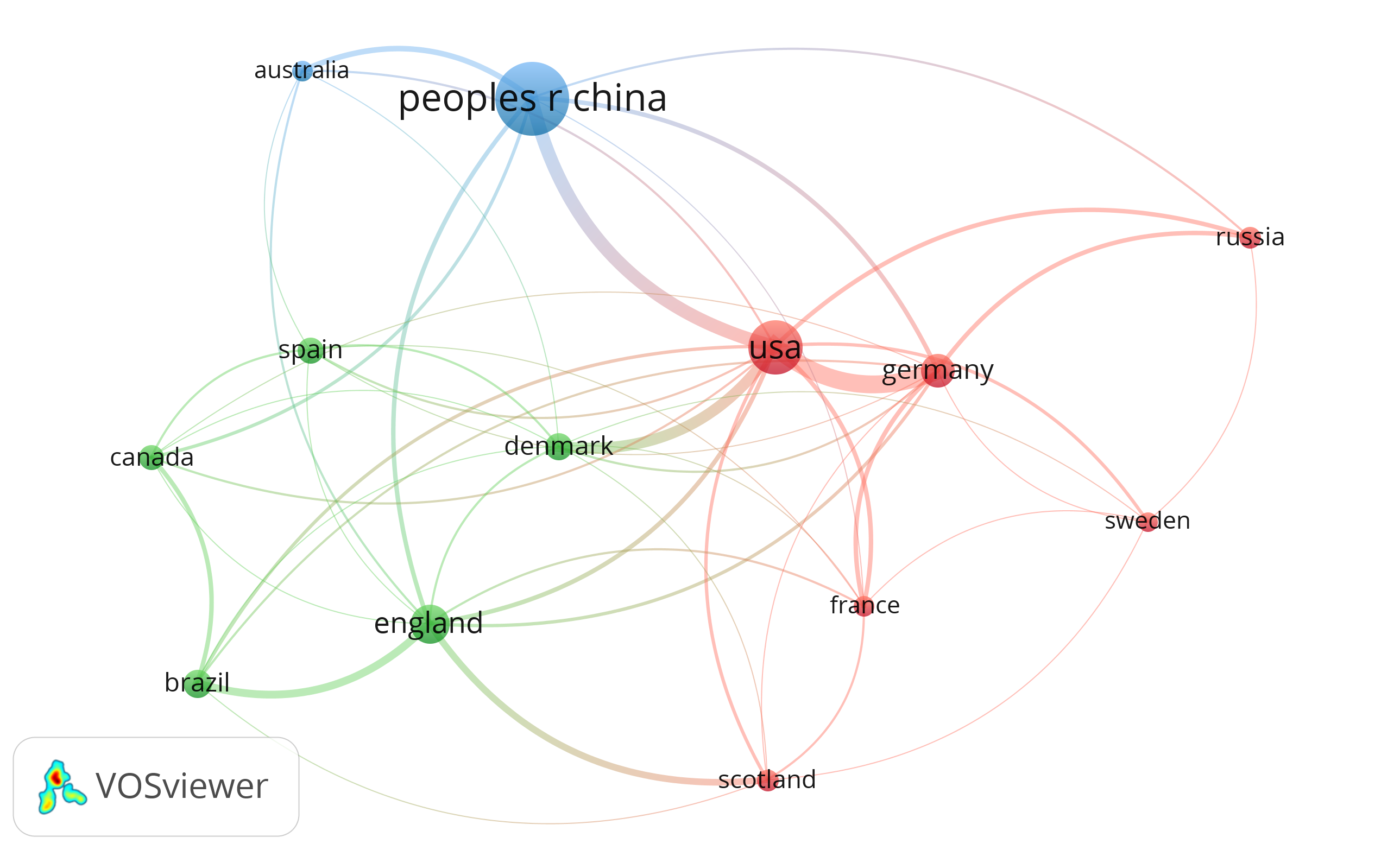

Supplement: Supplemental Information 2 [file peerj-12-18442-s002.png]
